# Correction: Mucosal effects for tenofovir 1% gel

**DOI:** 10.7554/eLife.07586

**Published:** 2015-03-24

**Authors:** Florian Hladik, Adam Burgener, Lamar Ballweber, Raphael Gottardo, Lucia Vojtech, Slim Fourati, James Y Dai, Mark J Cameron, Johanna Strobl, Sean M Hughes, Craig Hoesley, Philip Andrew, Sherri Johnson, Jeanna Piper, David R Friend, T Blake Ball, Ross D Cranston, Kenneth H Mayer, M Juliana McElrath, Ian McGowan

Hladik F, Burgener A, Ballweber L, Gottardo R, Vojtech L, Fourati S, Dai JY, Cameron MJ, Strobl J, Hughes SM, Hoesley C, Andrew P, Johnson S, Piper J, Friend DR, Ball TB, Cranston RD, Mayer KH, McElrath MJ, McGowan I. 2015. Muscosal effects for tenofovir 1% gel. *eLife* **4**:e04525. doi: 10.7554/eLife.04525. Published 3 February 2015

During the production process the CONSORT checklist and CONSORT flow chart were not attached to the article and thus were omitted from the published version. These items are now cited in the ethics section and added as Reporting standards.

In addition, the ‘Results’ have been moved so they appear before the ‘Materials and methods’ section. This formatting change has not affected the text or the meaning of the article.

This article has been corrected accordingly.

