# Supplementary material for: Mucosal effects of tenofovir 1% gel
Source: eLife. 2015 Feb 3;4:e04525. doi: 10.7554/eLife.04525 (PMC4391502; doi:10.7554/eLife.04525)
Supplement: Figure 3—source data 1. — DOI: http://dx.doi.org/10.7554/eLife.04525.010 [file elife04525s001.docx]

**Figure 3-source data 1. Pearson correlation coefficients of pre-processed microarray probe expression values between the three primary vaginal cell cultures**

|  | **No treatment** | | | **50 µM tenofovir** | | | **500 µM tenofovir** | | |  |
| --- | --- | --- | --- | --- | --- | --- | --- | --- | --- | --- |
|  | ρ_A,B_ | ρ_A,c_ | ρ_B,C_ | ρ_A,B_ | ρ_A,c_ | ρ_B,C_ | ρ_A,B_ | ρ_A,c_ | ρ_B,C_ |  |
| 1 d | 0.993 | 0.994 | 0.993 | 0.986 | 0.992 | 0.985 | 0.993 | 0.987 | 0.990 | 1 d |
| 4 d | 0.994 | 0.994 | 0.996 | 0.982 | 0.990 | 0.988 | 0.994 | 0.992 | 0.995 | 4 d |
| 7 d | 0.984 | 0.993 | 0.986 | 0.994 | 0.995 | 0.996 | 0.991 | 0.991 | 0.984 | 7 d |
| 14 d | 0.993 | 0.992 | 0.995 | 0.984 | 0.994 | 0.987 | 0.996 | 0.995 | 0.994 | 14 d |

A, B and C signify the three primary vaginal epithelial cell cultures; d, days of treatment
