# Supplementary material for: Mucosal effects of tenofovir 1% gel
Source: eLife. 2015 Feb 3;4:e04525. doi: 10.7554/eLife.04525 (PMC4391502; doi:10.7554/eLife.04525)
Supplement: Supplementary file 1. — Summary of effect sizes and statistical significance testing. DOI: http://dx.doi.org/10.7554/eLife.04525.016 [file elife04525s002.docx]

**Mucosal effects of tenofovir 1% gel (**Florian Hladik et al.)

**Statistics summary**

Figure 1C (colon vs. rectum, suppressed genes):

- Linear regression y = 0.3816x - 0.20, significantly non-zero slope? p <0.0001;
  - Slope 95% CI: 0.3323 to 0.4310
- Correlation spearman r = 0.4775, significant correlation? P<0.0001
  - 95% CI: 0.4051 to 0.5440

Figure S1 (colon v. rectum, induced genes):

- Linear regression y = 0.4921x - 0.10, significantly non-zero slope? p <0.0001;
  - Slope 95% CI: 0.3334 to 0.6507
- Correlation spearman r = 0.4270, significant correlation? P<0.0001
  - 95% CI: 0.2840 to 0.5514

Figure S2 (nonoxynol-9 vs tenofovir, strength of induction/suppression):

- Mann-Whitney (non-parametric, non-paired t test), two-tailed:
  - Suppression p<0.0001
    - Median, 95% CI of Median
      - nonoxynol-9 -0.6697, -0.7022 to -0.641
      - Tenofovir -0.986, -1.02 to -0.9575
  - Induction p = 0.0002
    - Median, 95% CI of Median
      - nonoxynol-9 0.7606, 0.7035-0.8749
      - Tenofovir 0.6635, 0.6297-0.6918

Figure 2C (microarray and ddPCR confirmations):

- Wilcoxon matched-pairs signed-rank one-tailed p-values done on PCR and microarray results:
  - Day 7 results divided by baseline results to give normalized day 7 value on individual PTID levels
  - Tests done on normalized day 7 value vs. normalized baseline value (i.e. 1)
- Down-regulated p-values multiplied by 6 (e.g. shown below)

|  | **Raw p-value** | **Adjusted p-value** | **Median fold change vs baseline, 95% CI of median** |
| --- | --- | --- | --- |
| DSP | <0.0001 |  | 0.2097, 0.1414-0.3532 |
| IL-10 | <0.0001 |  | 0.1538, 0.01303-0.4833 |
| TRIM5 | 0.0034 | 0.0204 | 0.5315, 0.2861-0.7129 |
| NFAT5 | 0.0002 | 0.0012 | 0.3014, 0.1698-0.3552 |
| TGFBRAP | <0.0001 |  | 0.3528, 0.2799-0.4526 |
| PAK2 | <0.0001 |  | 0.09436, 0.06016-0.1293 |
| PNPT1 | <0.0001 |  | 0.01634, 0.01036-0.1155 |

- Up-regulated p-values multiplied by 9

|  | **Raw p-value** | **Adjusted p-value** | **Median fold change vs baseline, 95% CI of median** |
| --- | --- | --- | --- |
| CCL19 | <0.0001 |  | 3.183, 1.988-7.946 |
| CCL21 | <0.0001 |  | 2.832, 1.654-5.406 |
| CCL23 | 0.0004 | 0.0036 | 2.322, 1.586-5.296 |
| CCR7 | 0.0002 | 0.0018 | 2.928, 1.442-16.57 |
| CD19 | <0.0001 |  | 3.875, 1.62-6.49 |
| CD7 | <0.0001 |  | 2.309, 1.618-18.83 |
| CXCL9 | <0.0001 |  | 4.017, 2.621-6.051 |
| MMP12 | <0.0001 |  | 2.419, 1.689-3.783 |
| SPINK4 | 0.0003 | 0.0027 | 3.275, 1.908-4.788 |

Figure 2D (immunohistochemistry):

- Paired t-test

|  | **P-value** | **Mean, 95% CI of mean** | **Mean fold change vs baseline, 95% CI of mean** |
| --- | --- | --- | --- |
| CD7  Log-trans | 0.0006 | Baseline: 1.759, 1.423-2.095  Day 7: 2.455, 2.16-2.749  (log transformed) | 0.6959, 0.3899-1.002 (in log_10_ units)  4.965, 2.454-10.05 (transformed back to normal units) |
| CD3  Log-trans | 0.0232 | Baseline: 2.54, 2.087-2.994  Day 7: 2.927, 2.661-3.194  (log transformed) | 0.3872, 0.06645-0.7080 (in log_10_ units)  2.439, 1.165-5.105 (transformed back to normal units) |
| UBD | 0.0071 | Baseline: 25.78, 20.26-31.3  Day 7: 34.82, 26.59-43.05 | 1.385, 1.089-1.680 |
| IL-10  Log-trans | 0.0168 | Baseline: 1.014, 0.5661-1.463  Day 7: 0.576, 0.1418-1.01  (log transformed) | -0.4384, -0.7771 to -0.09963 (in log_10_ units)  0.3644, 0.1671-0.7950 (transformed back to normal units) |

- Cells / mm^2^

|  | Mean cells / mm^2^, 95% CI of mean | |
| --- | --- | --- |
|  | Baseline | Day 7 |
| CD7 | 98.19, 17.52-178.9 | 408.5, 168.1-648.8 |
| CD3 | 854.8, 25.02-1685 | 1165, 422.4-1907 |
| IL-10 | 21.22, 2.235-40.19 | 9.574, -2.374 to 21.52 |

- Average mean staining intensity, 95% CI of mean

|  | Average mean staining intensity, 95% CI of mean | |
| --- | --- | --- |
|  | Baseline | Day 7 |
| UBD | 25.78, 20.26-31.30 | 34.82, 26.59-43.05 |

Figure 4B (HVE, ddPCR):

- Repeated measures ANOVA with Sidak’s multiple comparisons test

|  | **Comparisons** | **Adjusted p-value** | **Mean percent of no treatment, 95% CI of mean** | |
| --- | --- | --- | --- | --- |
| DSP | 500 µM day 1 vs. no treatment day 1  50 µM day 1 vs. no treatment day 1  500 µM day 7 vs. no treatment day 7  50 µM day 7 vs. no treatment day 7 | 0.0726  0.0951  0.0059  0.0046 | 86.95, 76.59-97.32  91.52, 86.23-96.81  22.15, 15.70-28.61  32.93, 23.98-41.88 | |
| IL10 | 500 µM day 1 vs. no treatment day 1  50 µM day 1 vs. no treatment day 1  500 µM day 7 vs. no treatment day 7  50 µM day 7 vs. no treatment day 7 | 0.0173  0.0350  0.0031  0.0024 | 35.05, 25.54-44.56  37.43, 20.47-54.38  1.40, 0.26-2.55  1.729, -0.91-4.37 | |
| KIAA0101  Log trans | 500 µM day 1 vs. no treatment day 1  50 µM day 1 vs. no treatment day 1  500 µM day 7 vs. no treatment day 7  50 µM day 7 vs. no treatment day 7 | 0.0158  0.0608  0.0054  0.0024 | 83.97, 50.94-117.0  39.97, 14.64-65.29  123.6, 89.92-157.3  71.09, 56.28-85.91  Log transformed | 691.4, 323.1-1479.1  251, 140.1-449.7  1721.9, 792.9-3741.1  513.9, 365.4-722.9  Transformed to normal units |

|  | **Samples** | **Mean copies per 1000 copies of HBB, 95% CI of mean** | |
| --- | --- | --- | --- |
|  |  | **Day 1** | **Day 7** |
| DSP | 500 µM  50 µM  no treatment | 1988, 1132-2843  2076, 1367-2785  2269, 1480-3058 | 881, 821.4-940.6  1330, 874.1-1786  4049, 3203-4895 |
| IL-10 | 500 µM  50 µM  no treatment | 20.52, 19.27-21.77  21.61, 17-26.23  59.8, 43.94-75.67 | 0.8672, 0.1493-1.585  1.152, -0.6941 to 2.998  62.7, 48.7-76.7 |
| KIAA0101  Log trans | 500 µM  50 µM  no treatment | 3.033, 2.714-3.353  2.593, 2.234-2.952  2.194, 1.873-2.514 | 3.605, 3.349-3.862  3.080, 3.006-3.154  2.369, 2.233-2.505 |

Figure 4C (HVE, IL10 ELISA):

- Repeated measures ANOVA with Sidak’s multiple comparisons test:

|  | **Adjusted p-value** | **Mean percent of no treatment, 95% CI of mean** |
| --- | --- | --- |
| 50 µM day 1 | 0.3324 | 95.25, 89.1-101.4 |
| 500 µM day 1 | 0.0381 | 79.5, 70-89 |
| 50 µM day 7 | 0.0068 | 75.5, 70.55-80.45 |
| 500 µM day 7 | 0.0002 | 6.75, 4.032-9.468 |

Figure 5A (mitochondria PNPT1):

- Repeated measures ANOVAs (paired, parametric) with Tukey post-tests: p < 0.0001

|  | **Comparisons** | **Adjusted p-value** | **Mean fold change vs. no treatment, 95% CI of mean** |
| --- | --- | --- | --- |
| Rectum | day 1 vs baseline  day 7 vs baseline  day 7 vs day 1 | 0.3964  <0.0001  0.0024 | 0.9209, 0.4547-1.387  0.09034, 0.0086-0.1721  0.2825, -0.0097-0.5748 |
| Colon | day 1 vs baseline  day 7 vs baseline  day 7 vs day 1 | 0.0019  <0.0001  0.4075 | 0.7341, 0.4516-1.017  0.5332, 0.3355-0.7310  0.9536, 0.6357-1.272 |

- Baseline colon vs rectum: paired two-tailed t-test p = 0.0041

|  | **Samples** | **Mean copies per 1000 copies of HBB, 95% CI of mean** |
| --- | --- | --- |
| Rectum | Baseline  day 1  day 7 | 140.2, 97.03-183.3  106, 47.47-164.6  9.686, -0.6125-19.98 |
| Colon | Baseline  day 1  day 7 | 76.37, 52.89-99.86  49.53, 25.9-73.16  40.41, 17.93-62.89 |

Figure 5B (mitochondria ATP6)

- Repeated measures ANOVAs (paired, parametric) with Tukey post-tests: p = 0.0002 (tenofovir), 0.4911 (nonoxynol-9)

|  | **Comparisons** | **Adjusted p-value** | **Mean fold change vs. no treatment, 95% CI of mean** |
| --- | --- | --- | --- |
| Tenofovir | day 1 vs baseline  day 7 vs baseline  day 7 vs day 1 | 0.0027  0.0002  0.6141 | 0.4484, 0.3163-0.5806  0.2490, 0.1505-0.3476  0.5881, 0.3774-0.7987 |
| Nonoxynol-9 | day 1 vs baseline  day 7 vs baseline  day 7 vs day 1 | ns  ns  ns | 0.9848, 0.9624-1.007  0.9964, 0.9870-1.006  1.013, 0.9936-1.032 |

- Baseline tenofovir vs N-9: unpaired, two-tailed t-test p 0.3439

|  | **Samples** | **Mean copies per 1000 copies of HBB, 95% CI of mean** |
| --- | --- | --- |
| Tenofovir | baseline  day 1  day 7 | 64446, 32476-96416  22500, 13977-31022  11701, 4485-18917 |
| Nonoxynol-9 | baseline  day 1  day 7 | 81490, 61003-101976  80699, 59630-101768  81203, 60797-101610 |

Figure 5c (mitochondria counts):

- Unpaired t-tests, Bonferroni adjusted (baseline vs. day 7)
  - Subject A: p = 0.0004
  - Subject B: p < 0.0001

|  | **Samples** | **Mean mitochondria / µm^2^, 95% CI of mean** |
| --- | --- | --- |
| Subject A | baseline  day 7 | 1.409, 1.211-1.607  0.8151, 0.6001-1.030 |
| Subject B | baseline  day 7 | 1.336, 1.124-1.548  0.3453, 0.2583-0.4323 |

Figure 5d (mitochondria area):

- Unpaired t-tests, Bonferroni adjusted (baseline vs. day 7)
  - Subject A: p = 0.196
  - Subject B: p < 0.0001

|  | **Samples** | **Mean mitochondrial area (µm^2^), 95% CI of mean** |
| --- | --- | --- |
| Subject A | baseline  day 7 | 0.05579, 0.05081-0.06076  0.06469, 0.05507-0.07432 |
| Subject B | baseline  day 7 | 0.05690, 0.05252-0.06127  0.07720, 0.07242-0.08197 |

Figure 5F (mitochondria gene expression in HVEs):

- Repeated measures ANOVA with Sidak’s multiple comparisons test

|  | **Comparisons** | **Adjusted p-value** | **Mean percent of no treatment, 95% CI of mean** |
| --- | --- | --- | --- |
| PNPT1 | 500 µM day 1 vs. no treatment day 1  50 µM day 1 vs. no treatment day 1  500 µM day 7 vs. no treatment day 7  50 µM day 7 vs. no treatment day 7 | 0.0517  0.0297  0.0061  0.0344 | 53.72, 29.59-77.86  70.99, 61.88-80.09  5.496, -1.183-12.18  60.85, 49.71-71.99 |
| ATP6 | 500 µM day 1 vs. no treatment day 1  50 µM day 1 vs. no treatment day 1  500 µM day 7 vs. no treatment day 7  50 µM day 7 vs. no treatment day 7 | 0.0408  0.0043  0.0484  0.1146 | 35.90, 26.00-45.81  55.34, 47.18-63.49  47.32, 26.48-68.16  67.20, 46.73-87.67 |

|  | **Samples** | **Mean copies per 1000 copies of HBB, 95% CI of mean** | |
| --- | --- | --- | --- |
|  |  | **Day 1** | **Day 7** |
| PNPT1 | 500 µM  50 µM  no treatment | 306.5, 172.2-440.8  405, 337.2-472.8  573.8, 443.6-703.9 | 25.27, -10.03-60.57  252.4, 191.8-312.9  420.8, 281.2-560.3 |
| ATP6 | 500 µM  50 µM  no treatment | 15290, 12847-17733  24760, 10840-38680  44015, 25842-62188 | 44410, 27231-61589  63225, 50776-75674  95688, 74178-117197 |
