# Supplementary figures and images for: Mucosal effects of tenofovir 1% gel
Source: eLife. 2015 Feb 3;4:e04525. doi: 10.7554/eLife.04525 (PMC4391502; doi:10.7554/eLife.04525)

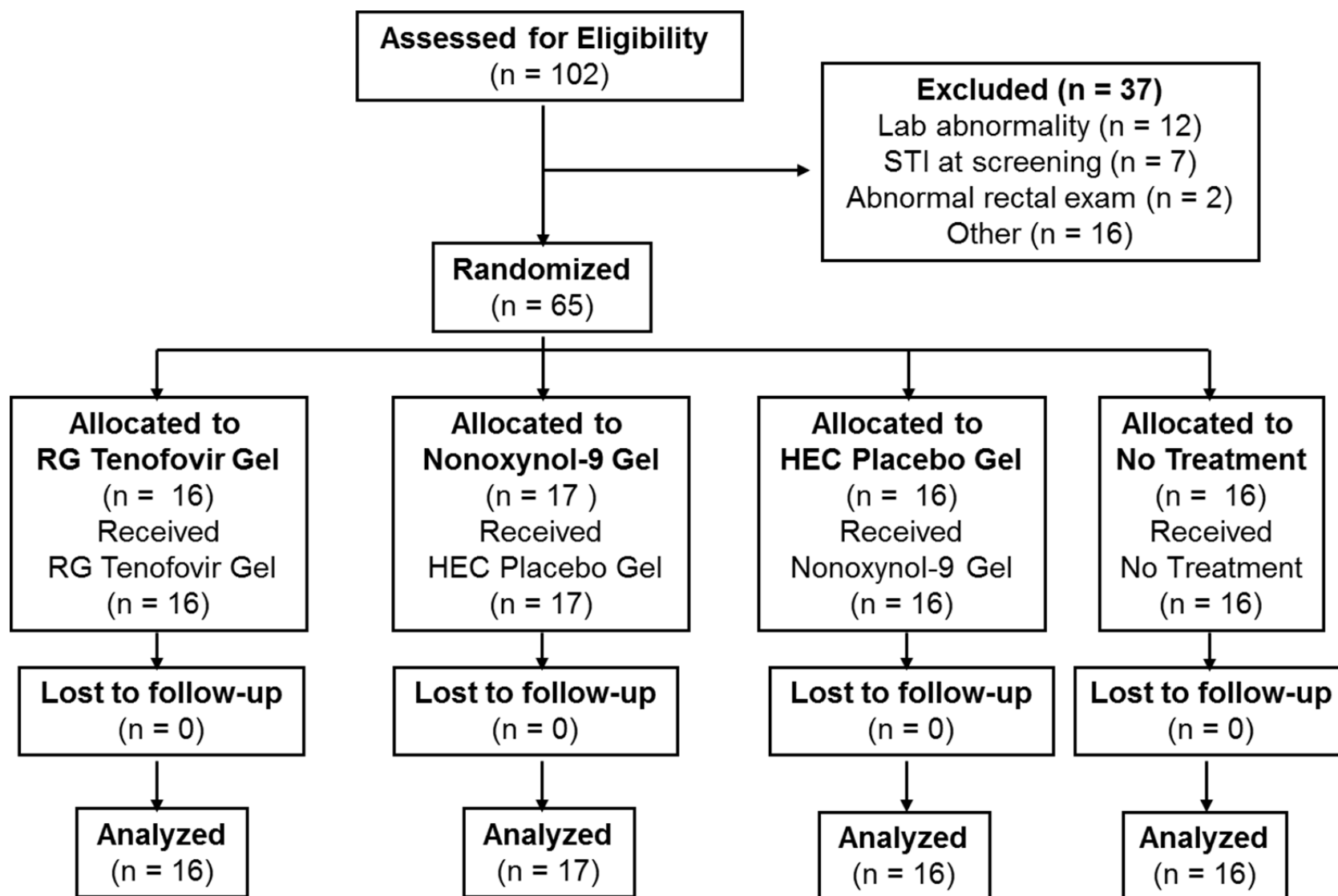

Supplement: Reporting standard 2. — DOI: http://dx.doi.org/10.7554/eLife.04525.021 [file elife04525s005.pdf]
